# Rabies Death Attributed to Exposure in Central America with Symptom Onset in a U.S. Detention Facility — Texas, 2013

**Published:** 2014-05-23

**Authors:** Ryan M. Wallace, Darlene Bhavnani, John Russell, Sherif Zaki, Atis Muehlenbachs, Kathryn Hayden-Pinneri, Ricardo Mena Aplícano, Leonard Peruski, Neil M. Vora, Diana Elson, Edith Lederman, Ben Leeson, Thomas McLaughlin, Steve Waterman, Maureen Fonseca-Ford, Jesse Blanton, Richard Franka, Andres Velasco-Villa, Michael Niezgoda, Lillian Orciari, Sergio Recuenco, Inger Damon, Cathleen Hanlon, Felix Jackson, Jessie Dyer, Ashutosh Wadhwa, Laura Robinson

**Affiliations:** 1 EIS officer, CDC; 2 Division of Global Migration and Quarantine, National Center for Emerging and Zoonotic Infectious Diseases, CDC; 3 Texas A&M Health Science Center, Christus Spohn Hospital, Corpus Christi, Texas; 4 Division of High-Consequence Pathogens and Pathology, National Center for Emerging and Zoonotic Infectious Diseases, CDC; 5 Harris County Medical Examiner’s Office, Texas; 6 Guatemala Ministry of Health; 7 Division of Global Health Protection, Center for Global Health, CDC; 8 Public Health, Safety, and Preparedness Unit, Immigrations and Customs Enforcement; 9 Texas Department of State Health Services

On June 7, 2013, a man was diagnosed in a Texas hospital with rabies. He had been detained in a U.S. detention facility during his infectious period. To identify persons exposed to rabies who might require rabies postexposure prophylaxis (PEP), CDC and the Texas Department of State Health Services (DSHS) conducted investigations at four detention facilities, one medical clinic, and two hospitals. In all, 25 of 742 persons assessed for rabies exposure were advised to receive PEP. Early diagnosis of rabies is essential for implementation of appropriate hospital infection control measures and for rapid assessment of potential contacts for PEP recommendations.

## Case Report

On May 9, 2013, a Guatemalan national aged 28 years, was apprehended by the U.S. Border Patrol. Seven days later, while in a U.S. Immigration and Customs Enforcement (ICE) detention facility, he experienced insomnia, anxiety, nausea, dysphagia, and multiple reported instances of hypersalivation and expectoration. He became increasingly agitated, developed tachycardia, and on May 18 was transported to a hospital emergency department where a computed tomography scan of the chest revealed pneumomediastinum. Shortly after assessment, he was transferred to a second hospital for surgery. Although the pneumomediastinum resolved without surgical intervention, his mental and respiratory status deteriorated. Initial laboratory analysis was notable for a peripheral leukocytosis of 27,700 cells/*μ*L (82% neutrophils) (normal cell count = 4,800–10,800/*μ*L). He was febrile, with a temperature of 103.6°F (39.8°C) and his mental and respiratory status deteriorated, prompting tracheal intubation. Labile blood pressure, hypersalivation, and an abnormal fear of drafts of fresh air were documented. A lumbar puncture was performed 11 days after symptom onset, yielding cerebrospinal fluid (CSF) with 18 white blood cells/*μ*L (normal = 0–5/*μ*L) and protein of 51 mg/dL (normal = 15–60 mg/dL). Magnetic resonance imaging of the brain showed no intracranial abnormalities.

Serum tested by enzyme-linked immunosorbent assay (ELISA) at a commercial laboratory detected rabies virus antibodies. Based on these results, the Milwaukee protocol (version 4.0),* an experimental rabies treatment plan, was initiated using ketamine, midazolam, insulin, amantadine, and nimodipine. Specimens were sent to CDC for confirmatory testing. Rabies virus-specific neutralizing and binding antibodies were detected in serum and CSF by the rapid fluorescent focus inhibition test (RFFIT) and indirect fluorescent antibody test (IFA), respectively.

Treatment was not successful, and the patient was pronounced brain-dead on June 11, hospital day 22, when life support was withdrawn. Nucleic acid amplicons from skin, saliva, and postmortem brain tissue were consistent with a canine rabies virus variant from Central America.

The patient had owned a dog in Guatemala, which died from unknown causes in 2011, but family members reported that they were unaware of any history of animal bites. No animal exposures were reported at the time of hospitalization and autopsy revealed no evidence of bite wounds. At the family’s request, the patient’s body was returned to his home in Guatemala after being embalmed using enhanced infection control measures under CDC consultation.

## Public Health Investigation

In studies of dogs, cats, and ferrets, shedding of rabies virus in saliva and tears occured up to 10 days before rabies symptom onset (1,2). Shedding of virus has not been adequately described for humans; therefore, the patient was considered potentially infectious from 14 days before symptom onset, which began with anxiety and insomnia on May 16 (Figure). Thirty-seven days after the start of the patient’s infectious period, CDC and the Texas DSHS began rabies contact investigations at four detention facilities where he hade been housed while under federal custody, a medical clinic at one detention facility, and two hospitals (A and B). All persons potentially in direct physical contact with the patient were considered for rabies risk assessment. Rabies PEP was recommended if there was a high probability of direct contact of saliva or tears with freshly broken skin or mucous membranes (2).

The patient was presumed to be in Mexico for the first 7 days of the potential infectious period. On day 8 he was apprehended while illegally entering the United States. During days 8–16 he was held at four detention facilities. The facilities hold males aged ≥18 years separate from juveniles and women. To accommodate routine disinfection of cells and cell capacity limitations, detainees are moved periodically. Records of detainee movements within a facility were not available; therefore, the patient had the opportunity to be housed with any adult male who was in the facility during the same time. On days 15 and 16 of his infectious period, the patient had multiple visits to a detention facility medical clinic. On day 16, the patient was transferred to hospital A and immediately referred to hospital B, where he received care until his death.

ICE records were used to construct a timeline for the period in which the patient was in the detention facilities. This timeline was then used to identify detainees and detention staff members who were in the facility with the patient. Age, sex, cumulative time with the patient, and facility-specific risk factors were used to develop a unique risk scoring system to prioritize rabies contact investigation activities. Risk scores for females and juveniles were zero, because they were not detained with the patient. Detainee contacts entering or exiting a facility with the patient received a risk score of 1 for each such event. Detainee contacts held in specific facilities where food and drink were likely to have been shared received a risk score of 2. Detainee contacts held in facilities when the patient was symptomatic received a risk score of 3. Detainees held in multiple facilities with the patient received higher cumulative risk scores.

The investigation identified 549 detainees with concordant detention timelines as the patient; 378 were adult males. A cumulative risk score of three or greater was the threshold for conducting a rabies risk assessment. Of the 68 detainees identified for risk assessment, 17 were still in ICE custody and were assessed by local or state health departments; one was recommended for PEP. The 51 remaining detainee contacts had since returned to one of four Latin American countries. The Pan-American Health Organization (PAHO) assisted in the notification process through the International Health Regulations.† The Ministry of Health in Guatemala, the patient’s country of origin, located 17 of 26 contacts; 13 were recommended for PEP. Officials from country B acknowledged receipt of the names of the 13 contacts recommended for risk assessment but declined to provide information on how investigations were conducted. Health officials from country C informed PAHO that they do not conduct contact investigations for prevention of human-to-human rabies transmission. The ministry of health for country D located all three contacts that were recommended for risk assessment; one was recommended for PEP (Table).

Texas DSHS and local health department staff members interviewed 185 law enforcement officers and contract staff members. Officers reported a physical altercation during the patient’s arrest. At the time of risk assessment (35 days after exposure), the officers could not recall if saliva from the patient made contact with open wounds or mucous membranes. PEP was recommended for the three officers involved in the patient’s apprehension. No other officers or contractors were recommended PEP.

Forty-four health-care workers from the detention facility medical unit and hospitals A and B were assessed for rabies virus exposures. Five healthcare workers received PEP because of potential exposure to saliva and tears during medical procedures. Two others received PEP at their own request.

### Discussion

Each year, approximately 55,000 persons worldwide die from rabies, a progressive encephalitic disease with a near 100% mortality rate. More than 98% of human rabies deaths are the result of transmission from the bite of a rabid dog, and nearly half of the world’s population currently live in countries in which canine rabies is endemic. In the United States, only 34 human rabies cases were reported during 2003–2013, of which 10 (29%) were attributed to rabid animal exposures abroad.

This investigation describes the first reported case of a person in federal custody while potentially infectious with rabies. Screening by ELISA detected rabies virus-specific binding antibodies for this patient, despite it not being a recommended assay for human rabies diagnosis (3). In the United States, antemortem rabies testing requires four specimens (serum, CSF, nuchal skin biopsy, and saliva), with serum and CSF tested by IFA and RFFIT. All persons potentially exposed to rabies virus should receive an individual risk assessment to determine if PEP is indicated. However, for rabies contact investigations occurring in large communal settings or multinational in scope, this might not be possible. In these circumstances, consultation with public health authorities regarding alternative options for risk assessment should be undertaken. To facilitate the contact investigation and prevent human-to-human rabies virus transmission, a unique risk scoring system was used for the first time to prioritize persons at highest risk for exposure in this specific instance.

Theoretically, human-to-human transmission can occur through exposure of mucous membranes or open wounds with saliva, tears, or nervous tissue from the infected patient. Detention facilities have increased potential for rabies virus exposures through close contact during confinement, as might have occurred when this patient began hypersalivating and expectorating in his cell (4). As a result of these circumstances, 15 of 37 (41%) detainees received PEP. In comparison, only three of 185 (2%) detention facility staff members and five of 44 (13%) medical staff members received PEP based on public health recommendations. Although human-to-human transmission of rabies is rare, the detention setting provides extensive opportunities for exposure.

Cremation is recommended for patients who have died from rabies. If cremation is not desired, the body should be permanently sealed in a closed casket without embalming. If the body must be prepared for public viewing, it should be embalmed using formalin with a concentration ≥2%, which has been shown to inactivate other enveloped RNA viruses (5,6). Although there is no evidence for aerosolization of rabies virus during routine embalming procedures, manipulation of the body and methods that use embalming fluids under pressure could potentially release infectious materials, particularly if organs and other tissues were removed during autopsy. The embalmer should use an N95 respirator, face shield, and puncture-resistant gloves in addition to standard infection control measures. Before dressing, the body should be disinfected with a 10% solution of sodium hypochlorite or equivalent disinfectant (7). Family members of rabies patients should avoid contact with the deceased body.

What is already known on this topic?

Each year approximately 55,000 persons worldwide die from rabies, a progressive encephalitic disease with a near 100% mortality rate. More than 98% of human rabies deaths are the result of transmission from the bite of a rabid dog. Nearly half of the world’s population currently lives in countries in which canine rabies is endemic. In the United States, only 34 human rabies cases were reported during 2003–2013, of which 10 (29%) were attributed to rabid animal exposures abroad.

What is added by this report?

In June 2013, a Guatemalan national aged 28 years was the first person known to have developed symptoms of rabies while in a United States detention facility. He developed insomnia, anxiety, nausea, dysphagia, and hypersalivation while detained. After hospitalization, his mental and respiratory status deteriorated, and 24 days after hospitalization he was declared brain dead. No animal exposures were reported, but phylogenetic analysis determined that the virus variant was associated with a canine variant found in Latin America. Of 742 detainee, enforcement officer, and medical contacts, 25 were recommended for and received postexposure prophylaxis.

What are the implications for public health practice?

Detention facility settings provide extensive opportunities for disease transmission, including rabies, and a complex network of potential disease contacts. Law enforcement and public health officials, when working cohesively, can quickly identify potentially exposed persons and provide life-saving medical recommendations. A diagnosis of rabies should be considered in patients hospitalized with unexplained acute progressive encephalitis, especially when the patient comes from a region with endemic rabies or has a known history of animal exposure.

Ministries of health responses to the International Health Regulations notification of potential human-to-human rabies transmission varied. Several countries attempted to locate all persons recommended for risk assessment; however, one country chose not to conduct an investigation and another did not provide details on how it responded. The outcome of these differing responses resulted in a low (54%) completion rate of confirmed rabies risk assessment among detainees. Human-to-human transmission of rabies is rare, and as such, public health programs must consider the costs and benefits of large-scale investigations. In countries where public health infrastructure can efficiently locate and treat contacts of rabies patients, this vaccine-preventable, fatal disease is usually thoroughly investigated. Development of streamlined approaches to human rabies contact investigations can enhance public health infrastructure and support best practices for use of public health and vaccine resources even in resource-limited countries.

## Figures and Tables

**FIGURE:**
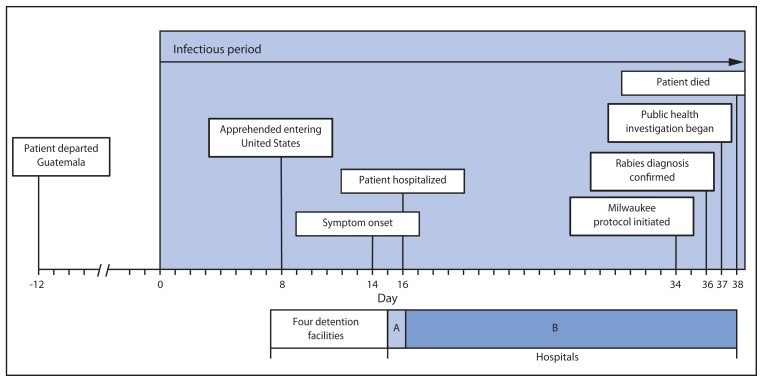
Timeline of rabies patient’s activities, by day of infectious period — Central America and Texas, 2013

**TABLE:** Rabies risk assessments and postexposure prophylaxis (PEP) recommendations associated with exposure to a patient with rabies in detention and medical facilities — Central America and Texas, 2013

Classification/Location	Potentially exposed	Recommended for rabies risk assessment	Risk assessments completed		PEP recommended	
	
			No.	(%)	No.	(%)
Detainees	378	68	37	(54)	15	(41)
U.S. custody	91	17	17	(100)	1	(6)
Guatemala	98	26	17	(65)	13	(76)
Country B	120	13	—*		—*	
Country C	56	4	—*		—*	
Country D	9	3	3	(100)	1	(33)
United States	4	0	—		—	
Enforcement officers and contract staff members	320†	320†	185	(58)	3	(2)
Medical staff members	44	44	38	(86)	5	(13)§
**Total**	**742**	**432**	**260**	**(60)**	**25**	**(10)**

* Country did not provide information on its activities in response to this event.

† Number of potentially exposed and assessment recommendations based on work shift listings; numbers are based on known or suspected contact with the index patient.

§ Five medical staff members were recommended for PEP. Two additional staff members received PEP at their own request, despite no evidence of a rabies exposure.

## Footnotes

* Available at http://www.mcw.edu/filelibrary/groups/pedsinfectiousdiseases/rabies/milwaukee_protocol_v4_20913.pdf.

† Additional information available at http://www.who.int/topics/international_health_regulations/en.
